# Synthesis of nano-octahedral MgO *via* a solvothermal-solid-decomposition method for the removal of methyl orange from aqueous solutions

**DOI:** 10.1039/c9ra10296e

**Published:** 2020-03-13

**Authors:** Xirui Yan, Zixin Tian, Wencai Peng, Jianshu Zhang, Yanbin Tong, Jun Li, Dekui Sun, Hui Ge, Jinli Zhang

**Affiliations:** School of Chemistry and Chemical Engineering, Shihezi University Shihezi 832003 Xinjiang China pengwencai@shzu.edu.cn junli@ipe.ac.cn; Key Laboratory for Green Processing of Chemical Engineering of Xinjiang Bingtuan Shihezi Xinjiang China; State Key Laboratory of Multiphase Complex Systems, Institute of Process Engineering, Chinese Academy of Sciences Beijing 100190 China; State Key Laboratory of Coal Conversion, Institute of Coal Chemistry, Chinese Academy of Sciences Taiyuan Shanxi China; Key Laboratory for Systems Bioengineering MOE, Tianjin University, Collaborative Innovation Centre of Chemical Science and Chemical Engineering (Tianjin) Tianjin 300072 China

## Abstract

Nano magnesium oxide has wide applications, and MgO with (111) facets has wider potential applications than MgO with (100) facets (*e.g.*, in catalysis and adsorption). However, nano MgO with (111) polar faces has not been studied throughly, so the preparation of nano-octahedral MgO (N-O-MgO) with eight exposed (111) facets remains a great challenge. Herein, we successfully synthesised N-O-MgO *via* an effective solvothermal-solid-decomposition method and studied its adsorption performance. The obtained N-O-MgO showed excellent performance (229.36 mg g^−1^) for methyl orange (MO). The adsorption follows the pseudo-second-order kinetic equation and the Langmuir isotherm model. The dimensionless parameter *R*_L_ (0.042) and Gibbs free energy Δ*G* (−6.538 kJ mol^−1^) revealed that the adsorption of MO on N-O-MgO was a spontaneous and feasible process. The adsorption of MO and methyl blue (MB) on N-O-MgO were studied to determine the adsorption sites. Based on these experiments and analysis, it was determined that the adsorption sites were magnesium ions and the adsorption mechanism was proposed to describe the adsorption process.

## Abstract 2

This article reports a facile nano-octahedral MgO synthetic method and its adsorption performance for methyl orange.

## Graphical abstract

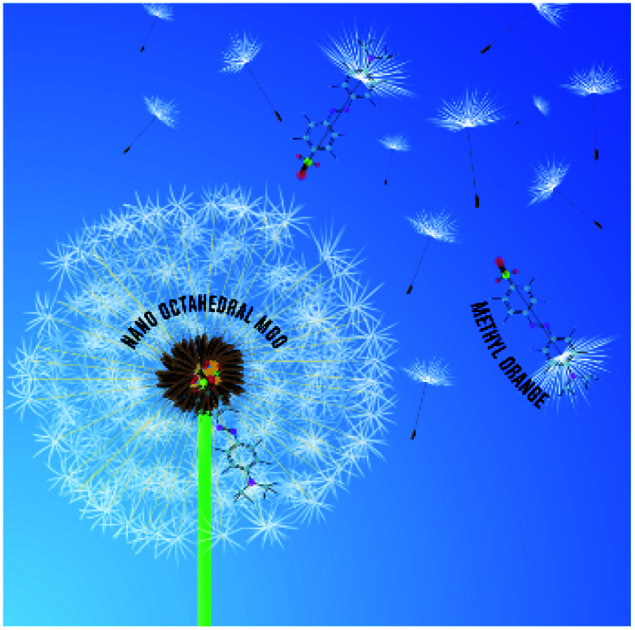

## 1. Introduction

Nano metal oxide materials have attracted numerous researchers due to their unique applications in the fields of energy and environment. As an important nano metal oxide, nano magnesium oxide (nano MgO) shows a wide range of applications, for *e.g.*, as a solid-base catalyst and carrier (for Claisen–Schmidt reaction,^1^ the preparation of Schiff bases,^2^ and the loading of nickel atoms^3^ for CO_2_ reforming of methane), as an adsorbent (for the removal of organic dyes such as methyl orange,^4^ heavy metal ions such as cadmium ions and lead ions,^5^ and fluoride ions^6^), and as a template and additive (for the preparation of graphene-like materials^7^ and the additive of CO_2_ adsorbent^8^). Nano MgO also shows optical properties^9^ and bactericidal properties.^10^ There are some methods used for the synthesis of nano MgO, which include precipitation,^11,12^ solid state reaction,^13^ microwave radiation method,^14^ and sol–gel route,.^15^ However, in these synthetic procedures,^9,10,15^ researchers have paid less attention to the crystal facet of nano MgO; MgO usually grows into a cubic structure with (100) facets.^16^

In the past few years, numerous studies have focused on MgO with the (111) polar facet due to its higher reactivity in catalysis^17,18^ and antibacterial^19^ applications. However, the reports about nano MgO with (111) polar facets and its properties are rare. Although a large MgO crystal can be mechanically cut and polished to obtain micro- or nano-scale materials, it would be difficult to control the facet that is exposed.^20^ It is an effective strategy to obtain MgO with the polar (111) facet by wet chemistry methods, which is often necessary to use additives in the reaction system to create a polar environment and to inhibit the rapid disappearance of the polar surface during the reaction. Ryan Richard *et al.*^17^ synthesised MgO (111) nanoplates by adding 4-methoxybenzyl alcohol in the reaction system, which has a strong interaction with the inorganic intermediate Mg(OH)–(OCH_3_). Jupille *et al.*^21^ obtained (111) facets *via* etching of cubic MgO smoke crystals using neutral water in wet conditions, which had an average particle size of 170 nm and a specific surface area of 5.5 m^2^ g^−1^. Hao *et al.*^19^ obtained MgO with (111) facets exposed *via* the solution combustion process of the mixture of magnesium nitrate and urea. Xie *et al.*^16,20^ prepared micro-scale octahedral MgO with 2.4 m^2^ g^−1^ specific surface area by decomposing magnesium nitrate in molten lithium nitrate at 400 °C. Further studies by Jeffrey D. Rimer^22^ discussed the influence of different factors on the morphology of MgO and demonstrated that the molten salt systems contributed to the formation of (111) facets in the decomposition of magnesium salts. For the molten salt systems, the crystal formed by the free movement of ions must grow to the microscale to reduce surface energy and maintain self-stability. Thus, the synthesis of nano-octahedral MgO with eight (111) facets exposed remains a huge challenge.

In this work, we first successfully prepared nano-scale octahedral MgO with eight (111) facets exposed *via* an effective solvothermal-solid-decomposition method and evaluated its adsorption performance using MO. The adsorption characteristics and mechanism were studied by a series of experiments and calculations, which included the simulation of different kinetic and isotherm model, and the determination of relevant thermodynamic parameters. Based on these analyses, the adsorption sites were determined and an adsorption mechanism for MO adsorption on N-O-MgO was proposed. These studies should be a useful reference for the preparation of materials and the exploration of adsorption mechanisms as well as for the preparation of nano materials with polar facets, the characteristics of nano materials with polar facet, and the exploration of adsorption mechanisms.

## 2. Experimental

### 2.1 Preparation of N-O-MgO

Magnesium nitrate (Mg(NO_3_)_2_·6H_2_O, AR); urea (AR) and ethanol (AR); methyl orange (MO) and methyl blue (MB) were purchased from Aladdin Co., Ltd; Tianjin Chemical Reagent Manufacturing Co., Ltd; Adamas Reagent Co., Ltd, respectively. All of them were used without further purification.

N-O-MgO was prepared *via* a solvothermal-solid-decomposition method. In a typical route, 2.56 g of Mg(NO_3_)_2_·6H_2_O was dissolved in 50 mL of ethanol and the homogeneous solution was transferred into a 100 mL Teflon bottle. Then, the mixture was thoroughly stirred after adding 6.00 g of urea into the solution. Subsequently, the Teflon bottle was placed in a stainless-steel vessel that was sealed and put into a temperature-controlled oven for solvothermal treatment at 200 °C for 24 h. After the heat treatment, fresh precipitates were collected, washed, and dried at 80 °C in air. N-O-MgO was produced by calcining the precipitates at 500 °C and continuing for 5 h.

### 2.2 Characterisation

The crystal structures of the obtained samples were examined by X-ray diffraction (XRD) on a D8 Advance X-ray diffractometer (Germany) using Cu Kα radiation.

The microscopic morphology of MgO was analysed by scanning electron microscopy (SEM) on a Hitachi S-4100 FE-SEM instrument (Japan) at 10 kV and transmission electron microscopy (TEM) on a Tecnai G2 F20 field emission transmission electron microscope (USA) at 200 kV.

N_2_ adsorption/desorption isotherm was recorded on a Micromeritics ASAP 2020 apparatus (USA) at 77 K for calculating the specific surface area and pore size distribution by the Brunauer–Emmett–Teller (BET) and Barrett–Joyner–Halenda (BJH) methods, respectively.

The vibrational characteristics of the obtained samples were recorded by a Fourier transform spectrometer (FT-IR; Agilent Cary 630, Agilent, America) in pressed KBr pellets.

The X-ray photoelectron spectrum (XPS) of N-O-MgO was obtained on a Kratos AMICUS spectrometer (SHIMADZU, JP) using Al Kα radiation. The binding energy of O element was calibrated relative to the carbon impurity with C 1s at 285 eV.

The concentrations of MO and MB in the aqueous solutions were measured using a Shimadzu UV-2550 UV-visible spectrometer (Japan).

### 2.3 Adsorption experiments

The batch adsorption experiments were carried out by mixing N-O-MgO particles with 20 mL aqueous MO solution under stirring at pH 6.0 and 303 K temperature at the rate of 150 rpm min^−1^. The three parameters were changed during the experiments: adsorbent amount (0.5–1.75 g L^−1^), contact time (5–150 min), and MO concentration (20–200 mg L^−1^). After the experiments, the mixture was separated and the ultimate concentration of MO was determined.

The adsorption capacity of MO on N-O-MgO and the removal ratio of MO were calculated using eqn (1) and (2).

1 *q*_e_ = (*C*_0_ − *C*_e_)*V*/*m*

2 *η* = 100%(*C*_0_ − *C*_e_)/*C*_0_

In which *q*_e_ (mg g^−1^) is the adsorption capacity of MO on N-O-MgO, *η* (%) represents the MO removal efficiency, *C*_0_ (mg L^−1^) is the MO initial concentration, *C*_e_ (mg L^−1^) is the adsorption equilibrium concentration of MO, *V* (L) is the MO solution volume, and *m* (g) is the weight of N-O-MgO particles.

The pseudo-first-order and pseudo-second-order (eqn (3) and (4)) kinetic models were simulated to describe the adsorption process of MO on N-O-MgO.

3 ln(*q*_e_ − *q*_*t*_) = ln *q*_e_ − *K*_1_*t*

4 *t*/*q*_*t*_ = 1/(*K*_2_*q*_e_^2^) + *t*/*q*_e_

where *q*_e_ and *q*_*t*_ (mg g^−1^) are the adsorption capacities of MO adsorbed on N-O-MgO at the equilibrium time and at a specific time *t* (min), *K*_1_ (min^−1^) is the pseudo-first-order model rate constant, and *K*_2_ (g mg^−1^ min^−1^) is the pseudo-second-order model rate constant.

The adsorption process of MO on the N-O-MgO particles was also simulated based on the Langmuir isotherm and Freundlich isotherm (eqn (5) and (6)). The dimensionless separation factor constant (*R*_L_, eqn (7)) was calculated to indicate the type of adsorption.

5 *q*_e_ = *q*_m_*K*_L_*C*_e_/(1 + *K*_L_*C*_e_)

6 *q*_e_ = *K*_F_*C*_e_^1/*n*^

7 *R*_L_ = 1/(1 + *K*_L_*C*_0_)

where *K*_L_ (L mg^−1^) is the Langmuir constant, *C*_e_ (mg L^−1^) is the adsorption equilibrium concentration of MO, *q*_e_ (mg g^−1^) is the adsorbed amount of MO on N-O-MgO at adsorption equilibrium, *q*_m_ (mg g^−1^) represents the maximum theoretical adsorption capacity, *K*_F_ (mg g^−1^) (L mg^−1^)^1/*n*^ is the Freundlich constant and *n* is the adsorption intensity. *R*_L_ > 1, *R*_L_ = 1, 0 < *R*_L_ < 1, and *R*_L_ = 0 indicate that the adsorption process is unfavourable, linear, favourable, and irreversible, respectively.

## 3. Results and discussion

### 3.1 Characterization of N-O-MgO

**Fig. 1:**
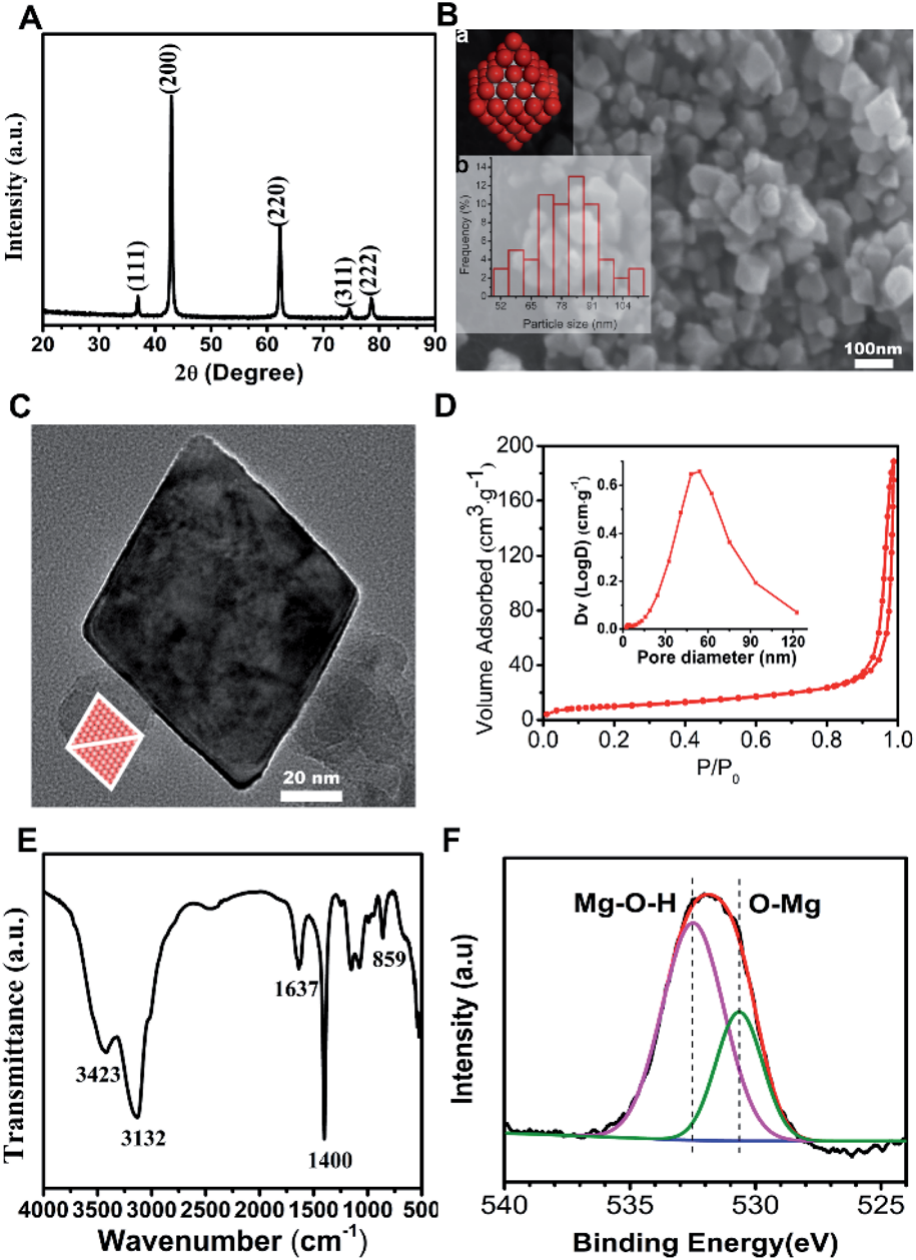
Characterisation of the synthesized MgO: (A) XRD pattern; (B) SEM image and corresponding (a, inset) model diagram of octahedral MgO and (b, inset) histogram of particle size distribution; (C) TEM image and model projected along the same crystal axis direction (inset); (D) N_2_ adsorption/desorption isotherm and pore size distribution (inset) of N-O-MgO. (E) FT-IR pattern; (F) O 1s XPS of N-O-MgO.

In this ethanol solvent system, urea molecules were a more advantageous ligand than water molecules for magnesium ions, which may inhibit the growth rate of MgO (111) facet and lead to octahedral growth under the decomposition process. Fig. 1(A) shows the XRD pattern of the products, where all the diffraction peaks agreed with the rock-salt structure MgO (JCPDS no. 45-0946), so the products were MgO. As seen in Fig. 1(B), the morphology of the products (MgO) was octahedral with an average particle size of 79.83 nm. This corresponded to the schematic model of rock-salt MgO with eight polar (111) facets exposed (inset a) and the histogram of particle size distribution is shown (inset b). TEM was carried out to characterize the exposed facets of the MgO octahedral. Fig. 1(C) presents the TEM image along the [110] zone axis, in which the octahedron MgO appeared to be a diamond. The diamond model (inset) was drawn by projecting the octahedron along the [110] direction, which was in good agreement with the TEM image. These analyses proved that the as-prepared MgO was nano-octahedral with eight exposed (111) polar facets. The N_2_ adsorption/desorption isotherm and pore size distribution curve (inset) of N-O-MgO are shown in Fig. 1(D). The N-O-MgO isotherm showed type-IV characteristics with a significant H3 loop. The formation of pores in N-O-MgO was a result of the aggregation of the particles. The BET specific surface area was 36.84 m^2^ g^−1^, which was higher than that of micro-scale octahedral MgO (2.4 m^2^ g^−1^).^16^Fig. 1(E) shows the FT-IR pattern of N-O-MgO. The broad bands located at 3420 cm^−1^ and 1637 cm^−1^ correspond to water molecules or hydroxyl groups.^23^ The peak located at 3132 cm^−1^ may result from the hydrogen bonds. The peaks at 1400 cm^−1^ and 859 cm^−1^ were assigned to magnesium–oxygen (Mg–O) group or magnesium–hydroxyl (Mg–OH) group.^24^ The FT-IR spectrum revealed abundant hydroxyl groups on N-O-MgO surfaces. The O element of N-O-MgO was investigated by X-ray photoelectron spectroscopy. The O 1s XPS is shown in Fig. 1(F), in which the O 1s spectrum is divided into two peaks. The binding energies of 530 and 532 eV corresponded to the lattice oxygen^25^ (Mg–O) and magnesium hydroxyl group (Mg–O–H), respectively. The result also indicated abundant hydroxyl groups on N-O-MgO surfaces, which was in accordance with the FT-IR analysis.

### 3.2 Adsorption experiment

#### 3.2.1 Effect of adsorbent amount

**Fig. 2:**
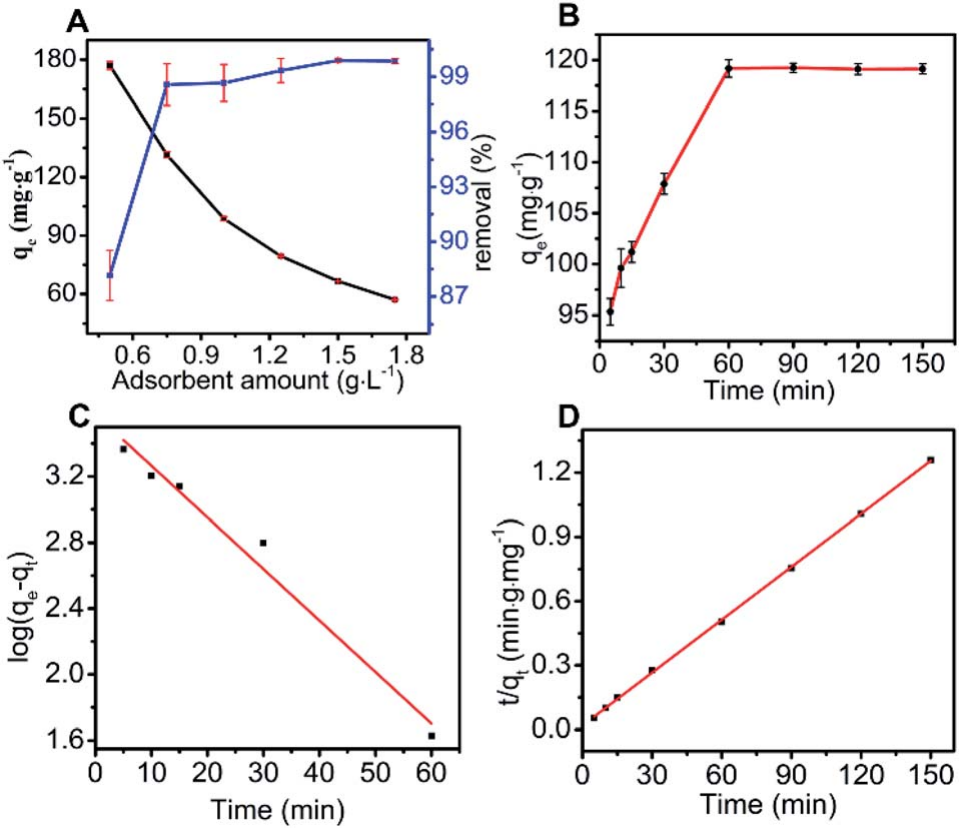
Effect of (A) adsorbent amount and (B) contact time (adsorbent amount = 0.75 g L^−1^, pH = 6, *T* = 303 K, *t* = 150 min) on the removal of MO by N-O-MgO. Derived (C) pseudo-first and (D) pseudo-second-order kinetic models.

As an important parameter, adsorbent amount was investigated for the removal of MO under equilibrium conditions, as shown in Fig. 2(A). As N-O-MgO concentration increased from 0.5 to 1.75 g L^−1^, its adsorption capacity gradually decreased from 176.30 to 56.28 mg g^−1^, likely due to decrease in the utilisation rate of the adsorption sites on increasing the amount of N-O-MgO.^26^

The initial removal percentage was over 81.85% and reached 99.89% when the adsorbent amount was 0.75 g L^−1^; then, the removal rate increased slowly. This was explained by the increase in adsorption sites for higher N-O-MgO amounts, leading to the increase in removal rate.^27^

#### 3.2.2 Effect of contact time and adsorption kinetics

The contact time remarkably influenced the adsorption of MO on N-O-MgO, as shown in Fig. 2(B). For the adsorption of MO on N-O-MgO, the adsorption was fast in the first 5 min, implying that N-O-MgO could rapidly adsorb MO molecules from the aqueous solution; the adsorption equilibrium was reached after 60 min, where the adsorption amount was 119.42 mg g^−1^.

The adsorption kinetic behaviour of MO on N-O-MgO was simulated using pseudo-first-order and pseudo-second-order kinetic models. The fitting results and relevant parameters are shown in Fig. 2(C), (D) and Table 1, respectively. Compared to the simulation by pseudo-first-order model, the calculation results using the pseudo-second-order model were closer to the experimental data and its correlation coefficient *R*^2^ was higher than 0.999. This indicated that the adsorption of MO on N-O-MgO can be more adequately described by the pseudo-second-order kinetic model. For this adsorption process, the overall adsorption rate of MO was controlled by the chemical process through ion exchange between MO and N-O-MgO.^28^ A detailed explanation of the adsorption process is provided in Section 3.3.

**Table 1:** Kinetic parameters for the adsorption of MO on N-O-MgO

*C*, mg L^−1^	*q*_e,exp_, mg g^−1^	Pseudo-first-order model			Pseudo-second-order model		
		*q*_e,cal_, mg g^−1^	*K*_1_, g mg^−1^ min^−1^	*R* ^2^	*q*_e,cal_, mg g^−1^	*K*_1_, g mg^−1^ min^−1^	*R* ^2^
100	119.42	30.62	0.0226	0.8943	121.36	0.0037	0.9997

#### 3.2.3 Effect of pH

**Fig. 3:**
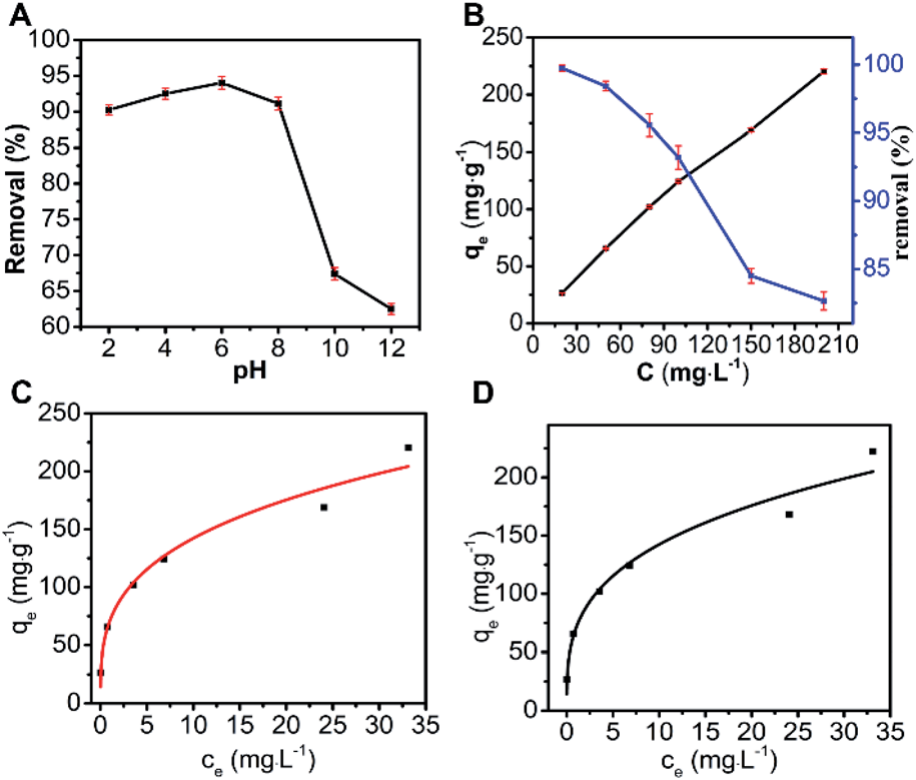
Effect of (A) pH and (B) MO concentration (adsorbent amount = 0.75 g L^−1^, pH = 6, *T* = 303 K, *t* = 150 min) on the removal of MO by N-O-MgO; (C) Langmuir isotherm; (D) Freundlich isotherm.

The effect of pH in the range from 2 to 12 on the adsorption of MO on N-O-MgO is shown in Fig. 3(A). The pH was adjusted with 0.1 M HCl and 0.1 M NaOH solutions, and the pH of each solution was measured by using a laboratory scale pH meter (PHS-25). As the pH increased from 2 to 6, the adsorption capacity of N-O-MgO slowly increased from 120 to 124, and the highest adsorption capacity was obtained at pH = 6. As the pH increased from 6 to 12, the adsorption capacity of N-O-MgO evidently decreased. With the increase in the concentration of H^+^, the inhibition of electrostatic interaction between the –SO_3_^−^ groups of MO and the positive charge of the N-O-MgO surface was weak. With the increase in the concentration of OH^−^, it competed with the –SO_3_^−^ groups of MO for the adsorption sites on the adsorbent surface.^29,30^ The analysis indicated that the electrostatic interaction might be the adsorption mechanism for MO adsorption on N-O-MgO.

#### 3.2.4 Effect of MO concentration and adsorption isotherms

The effect of MO concentration on its adsorption on N-O-MgO was investigated, as shown in Fig. 3(B). As the concentration increased from 20 to 200 mg L^−1^, the adsorption capacity of N-O-MgO gradually increased. This indicated that N-O-MgO had good adsorption performance for MO molecules in aqueous solution.

The fitting result and relevant parameters for MO adsorption using the Langmuir and Freundlich isotherm models are presented in Fig. 3(C), (D) and Table 2, respectively. According to the fitting result, the adsorption parameters fitted well with both the Langmuir isotherm (*R*^2^ = 0.9520) and Freundlich isotherm (*R*^2^ = 0.9594). Here, we used the Langmuir isotherm model to describe the adsorption process for MO. For the Langmuir isotherm model, the adsorption of MO on N-O-MgO was monolayer. The *R*_L_ value was 0.042 by calculation, implying favourable adsorption of MO on N-O-MgO.

**Table 2:** Isotherm models' parameters for the adsorption of MO on N-O-MgO

Langmuir isotherm				Freundlich isotherm		
*q*_m_, (mg g^−1^)	*K*_L_, (L mg^−1^)	*R* ^2^	*R*_L_	*K*_F_, (mg g^−1^) (L mg^−1^)^1/*n*^	*n*	*R* ^2^
229.36	0.2280	0.9520	0.042	70.69	3.2873	0.9594

#### 3.2.5 Recyclability

**Fig. 4:**
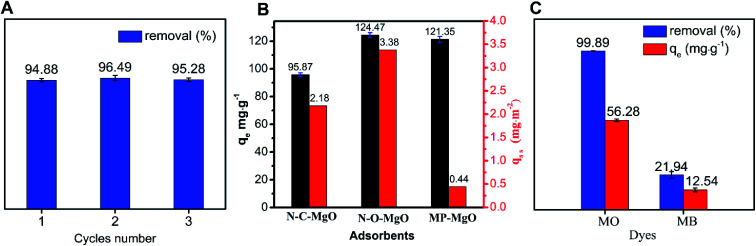
(A) Regeneration of N-O-MgO adsorbent; (B) comparison of the adsorption capacity and adsorption capacity per unit specific surface area (*q*_s s_) for MO adsorption on N-C-MgO, MP-MgO, and N-O-MgO (MO concentration = 100 mg L^−1^, pH = 6, *T* = 303 K, *t* = 60 min, N-O-MgO dosage = 0.75 g L^−1^); (C) adsorption of MO and MB on N-O-MgO (dyes' concentration = 100 mg L^−1^, *T* = 303 K, pH = 6, *t* = 150 min, N-O-MgO dosage = 1.75 g L^−1^).

The reusability of an adsorbent is essential for its practical application. The results of N-O-MgO regeneration are shown in Fig. 4(A). The removal rate of the adsorbent after two cycles was still over 94%. The results implied that N-O-MgO was an efficient adsorbent with excellent reusability. Moreover, the regeneration of N-O-MgO was achieved through simple calcination for 3 h at 500 °C after separation.

#### 3.2.6 Comparison of synthetic MgO and N-O-MgO

For the comparative experiment, our purpose was to study the adsorption performance of (100) and (111) facets of MgO. As shown in Fig. 4(B), the adsorption capacity and adsorption capacity per unit specific surface area for MO was examined for both synthetic MgO (N-C-MgO, MP-MgO) and N-O-MgO. N-C-MgO with the (100) facet exposed was prepared *via* a sol–gel method, which has a particle size distribution in the range of 20–40 nm. In the typical route, 1.00 g P123 was completely dissolved in 60 mL of ethanol and then, the mixture was thoroughly stirred after adding 0.76 g magnesium ethanol and 1.5 mL HNO_3_ for 5 h. The mixture solution was dried at 60 °C and calcined at 500 °C for 5 h. MP-MgO was prepared *via* a hydrothermal method previously reported by our research group,^31^ which has a particle size distribution in the range of 20–80 μm. Although N-C-MgO and MP-MgO have a higher specific surface area (43.90 m^2^ g^−1^ and 278 m^2^ g^−1^) than N-O-MgO (36.84 m^2^ g^−1^), the adsorption capacity of MO on cube-MgO (95.86 mg g^−1^) was lower than that on N-O-MgO; the adsorption capacity of MO on MP-MgO was closed to that of N-O-MgO. Further, for the adsorption capacity per unit specific surface area, the order was N-O-MgO (3.38 mg m^−2^) > cube-MgO (2.18 mg m^−2^) > MP-MgO (0.44 mg m^−2^). Based on the contrast of three samples *via* adsorption capacity and adsorption capacity per specific surface area, N-O-MgO had a better adsorption performance, indicating that the (111) facet has higher activity than the (100) facet. For adsorption, in addition to the influence of the crystal plane, the diffusion influence of MO molecules might be another important factor. As MO is a long chain molecule and MP-MgO is a porous material, its low adsorption capacity might be attributed to diffusion restriction.

### 3.3 Adsorption mechanism

#### 3.3.1 Determination of the adsorption sites

**Scheme 1:**
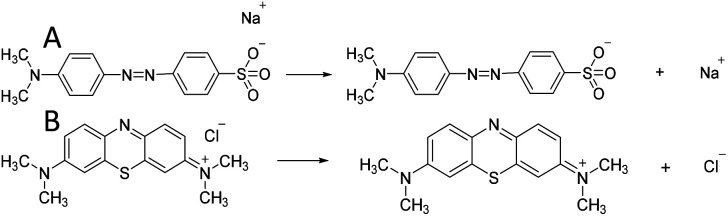
Ionisation equations of (A) MO and (B) MB in aqueous solution.

Adsorption experiments were carried out for two typical dyes (anionic MO and cationic MB) using N-O-MgO as the adsorbent to determine its adsorption sites. The results are presented in Fig. 4(C). The adsorption capacity and removal rate of MO on N-O-MgO were much higher than those of MB. The differences in adsorption were caused by the characteristics of N-O-MgO. In the outermost layer of N-O-MgO, whether it was positively or negatively charged, a hydroxyl layer was formed in the aqueous solution, which acted as the new outermost layer.^32,33^ Compared to having only hydrogen atoms at the outermost, the hydroxyl dropped much more easily, forming adsorption sites with positive charge (Mg^2+^). Thus, the –SO_3_^−^ groups of MO with negative charge could be better adsorbed on the adsorption sites of N-O-MgO than the positively charged MB by ion exchange. The ionisation of MO and MB molecules in aqueous solution is shown in Scheme 1.

#### 3.3.2 Utilisation ratio of adsorption sites

The utilisation ratio of N-O-MgO adsorption sites was calculated based on the theoretical Langmuir isotherm for saturated adsorption. The utilization ratio was the value of the adsorbed number of MO molecules over the amount of outmost layer oxygen ions on N-O-MgO due to the formation of the hydroxyl layer elaborated in Section 3.3.1.

① The distance (*d*, eqn (8)) between two oxygen ions and the area (*S*_2_, eqn (9)) of a diamond composed by four adjacent oxygen ions on the surface of N-O-MgO were calculated based on rock-salt contracture.

8 2*a*^2^ = (2*d*)^2^

9 *S*_2_ = *d*^2^√3/2

② Number of atoms forming a diamond (*n*, eqn (10)):

10 *n* = 2/6 + 2/3 = 1

③ Calculation of the utilisation ratio (eqn (11)–(13)) of the N-O-MgO adsorption sites.

The amount of outmost layer oxygen ions on the N-O-MgO (111) facet:

11 *N*_1_ = *S*_1_/*S*_2_

The number of MO molecules adsorbed on N-O-MgO:

12 *N*_2_ = (*q*_e_*A*_a_)/*M*

The utilisation ratio of adsorption sites:

13 *R* = *N*_2_/*N*_1_

In which *a* (0.4211 nm) is the cell constant, *d* (nm) is the distance between two magnesium on the (111) facet, *S*_2_ (nm^2^) is the area of a diamond composed by four adjacent Mg atoms on the (111) facet, *S*_1_ (36.84 m^2^) is the specific surface area of a unit mass of N-O-MgO particles, *N*_1_ is the amount of outmost layer O ions on N-O-MgO per unit mass, *N*_2_ is the adsorption number of MO molecules on N-O-MgO, *R* is the utilisation ratio of adsorption sites on N-O-MgO, *q*_e_ (mg g^−1^) is the amount of adsorbed MO molecules on the adsorbent surface at equilibrium, *A*_a_ (6.022 × 10^23^ mol^−1^) is the Avogadro's constant, and *M* (327.36 g mol^−1^) is the molar mass of MO.

The calculations indicate that the utilisation ratio of the adsorption sites on N-O-MgO was 91.70%, indicating that MO molecules effectively formed a monolayer on N-O-MgO.

#### 3.3.3 Adsorption mechanism

An equation for MO adsorption on N-O-MgO was proposed, which is eqn (14). To evaluate such a process, the thermodynamic parameters Gibbs free energy was calculated at 303 K. The distribution coefficient (*K*) and Gibbs free energy (Δ*G*) were determined using eqn (15) and (16).

14 Dye-SO_3_Na + N-O-MgO → dye-SO_3_–Mg–MgO-O-N + NaOH

15 *K* = (*C*_0_ − *C*_e_)/*C*_0_

16 Δ*G* = −*RT* ln *K*

In which *C*_0_ (mg L^−1^), *C*_e_ (mg L^−1^), *R* (8.314 J mol^−1^ K^−1^), and *T* (303 K) are the initial concentration of MO, equilibrium concentration of MO, gas constant, and absolute temperature, respectively.

Δ*G* was calculated as −6.538 kJ mol^−1^, demonstrating that the adsorption process of MO on N-O-MgO is spontaneous and feasible, which is in accordance with the analysis of the Langmuir isotherm model.

**Fig. 5:**
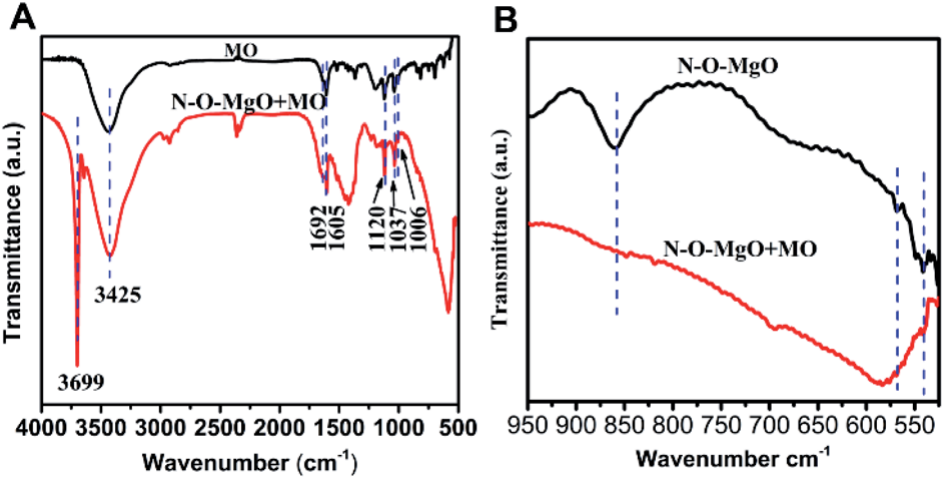
(A) The FT-IR spectra of MO and N-O-MgO after MO adsorption; (B) the FT-IR spectra of N-O-MgO before and after MO adsorption at 525–950 cm^−1^.

In order to further describe the adsorption process, MO and N-O-MgO after MO adsorption were characterized by FT-IR spectroscopy. Fig. 5(A) shows the FTIR spectra of MO and N-O-MgO after MO adsorption. The FT-IR spectra peaks located at 1605 cm^−1^, 1120 cm^−1^, 1692 cm^−1^ and 1037 cm^−1^, and 1006 cm^−1^ were attributed to phenyl groups,^30^ C–N group,^34^ and sulfonic acid group (–SO_3_^−^),^35^ and C–H in-plane of benzene rings with bending vibration, respectively. The presence of FT-IR peaks of MO on N-O-MgO after MO adsorption confirmed that the MO molecules were adsorbed on the surface of N-O-MgO. The peaks located at 3699 cm^−1^ and at 3425 cm^−1^ were attributed to the stretching of free- and associated-hydroxyl groups, which may be caused by the formation of magnesium hydroxide (Mg(OH)_2_) and physiosorbed water^36^ on the surface of N-O-MgO in the adsorption process. Compared with the FT-IR spectra of N-O-MgO shown in Fig. 1(E), the strength of hydroxyl group peaks of N-O-MgO after MO adsorption became weak, indicating that –SO_3_^−^ groups of MO were substituted with the hydroxyl groups in the MO adsorption process.^30,37^ The FT-IR spectra of N-O-MgO before and after MO adsorption at 525–950 cm^−1^ were shown in Fig. 5(B). The peaks of Mg–O–Mg and O–Mg–O (fingerprint area) of N-O-MgO after MO adsorption disappeared or shifted, further confirming that the –SO_3_^−^ groups of MO were connected with Mg^2+^ of the N-O-MgO surface.^30,38^

**Scheme 2:**
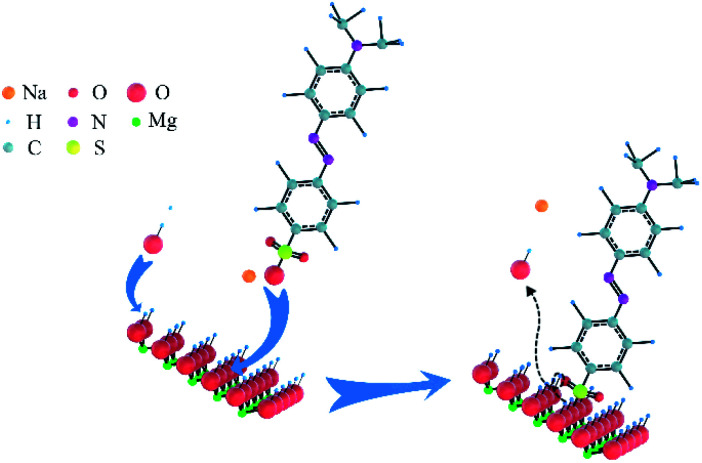
Adsorption mechanism of MO on N-O-MgO. Ionic radius of oxygen is larger than the covalent radius of oxygen. The balls of Na and Mg are ionic radius. The balls of H, N, C, and S are the covalent radius.

Based on these analyses above, the adsorption mechanism was proposed in Scheme 2. As shown in Scheme 1(A), the exposed –SO_3_^−^ groups of MO and sodium ions were formed *via* the ionisation of MO molecules in aqueous solution. For the adsorption process, a coordination hydroxyl group of a magnesium ion broke away from its original position and left a vacancy to form OH^−^ in the aqueous solution; then, a –SO_3_^−^ group of MO with negative charge exchanged into the vacancy and bonded the magnesium ion by ion exchange and electrostatic interaction. As given in the analysis in Section 3.3.2, the utilisation ratio of adsorption sites on N-O-MgO reached 91.70%, indicating that the exchange of hydroxyls and –SO_3_^−^ groups of MO were ongoing until the adsorption of MO molecules on N-O-MgO formed a monolayer.

## 4. Conclusions

In summary, we have obtained N-O-MgO particles *via* an effective solvothermal-solid-decomposition method and used it as an adsorbent for MO adsorption to study its adsorption performance. The adsorption process of MO on N-O-MgO in aqueous solution was systematically studied under different experimental conditions (adsorbent amount, contact time, MO concentration, and pH). The results revealed that the pseudo-second-order kinetic model and monolayer adsorption precisely described the spontaneous and feasible adsorption process, which was analysed by the *R*_L_ and thermodynamic analyses. N-O-MgO had excellent recyclability and higher activity than N-C-MgO and MP-MgO with (100) facets exposed. The adsorption sites were identified as magnesium ions that were effectively utilised on the adsorption process. A possible adsorption mechanism was proposed based on the analysis of experiments, calculation, and characterization. The results of this study may be a reference for the preparation of nanomaterials with polar facets and adsorption mechanism with, for example, different MgO morphologies or even different materials.

## Conflicts of interest

There are no conflicts to declare.

## Supplementary Material
